# First report of *Ovomermis sinensis* (Nematoda: Mermithidae) parasitizing fall armyworm *Spodoptera frugiperda* (Lepidoptera: Noctuidae) in China

**DOI:** 10.21307/jofnem-2020-050

**Published:** 2020-05-18

**Authors:** Bingjiao Sun, Fen Li, Xiaorui He, Fengqin Cao, Elizabeth Bandason, David Shapiro-Ilan, Weibin Ruan, Shaoying Wu

**Affiliations:** 1 College of Life Sciences, Nankai University, Tianjin 300071, China; 2 Key Laboratory of Green Prevention and Control of Tropical Plant Diseases and Pests, Ministry of Education, College of Plant Protection, Hainan University, Haikou 570228, China; 3 Lilongwe University of Agriculture and Natural Resources, Bunda Campus, P.O. Box 219, Lilongwe, Malawi; 4 USDA-ARS, SEFTNRL, Byron, GA 31008

**Keywords:** Biological control, Monoxenous obligate parasites, Fall armyworm, Mermithidae, Insect parasitism

## Abstract

*Spodoptera frugiperda* invaded China in the end of 2018 and has caused severe damage to maize and other crops. Several *S. frugiperda* naturally parasitized by nematodes were observed in Hainan Province, China. The morphological characteristics based on the results of scanning electron microscopy indicated that the nematode belongs to the family Mermithidae. Additionally, coding sequences for the 18 S and 28 S rDNA were amplified from the nematode genome, and phylogenetic analysis revealed that the nematode belongs to *Ovomermis sinensis*, a known entomoparasitic nematode. Our finding is the first record that *S. frugiperda* was naturally parasitized by *O. sinensis*. The results of this study are of great significance for potential biological control of *S. frugiperda* by indigenous natural beneficial organisms, i.e. *O. sinensis* within an integrated pest management system.

*Spodoptera frugiperda* (J. E. Smith), fall armyworm (FAW) is a pest native to tropical and subtropical regions of the America and widely distributed throughout the American continents (Todd and Poole, 1998). The pest, which is indigenous, is highly polyphagous, causing economic damage in various crops such as maize, beans, cotton, and sorghum (Day et al., 2017). Due to its voracity, high dispersal ability, wide host range, and high fecundity, it has already invaded many countries in Africa and Asia and is causing substantial yield losses (Baudron et al., 2019). FAW causes up to $16 billion in crop losses across Africa annually (Harrison et al., 2019). FAW invaded Yunnan province, China in late 2018. Since then FAW has dramatically spread in China (Zhang et al., 2019; Li et al., 2019), and was recorded in 1 million hectares from 26 provinces in China by December 2019.

Due to intensive pesticide application, FAW has developed resistance to a variety of chemical pesticides in several countries (Yu et al., 2003; Zhao et al., 2019), i.e. substantially high levels of resistance to omethoate was observed in FAW captured in China (Zhao et al., 2019). Based on the adverse effects of some chemicals on human health, the environment and living organisms, researchers are focusing on potential biological control agents (Harrison et al., 2019). FAW are attacked by various natural biocontrol agents such as parasitoids (López et al., 2018), bacteria (del Valle Loto et al., 2019), fungi (Shylesha et al., 2018), nematodes (Ruiz-Nájera et al., 2013; Viteri et al., 2018), and virus (Souza et al., 2019). There is a dearth of information on natural enemies of FAW, in China, particularly because the insect only invaded the region within the past year. It is high time to develop biological control methods for FAW in China. Although assessing the virulence of commercial biological control agents is a very important way to control FAW from the perspective of environmental protection, there is also a great need to understand, promote, and maximize the effectiveness of indigenous populations of natural enemies. Nematodes are the most abundant metazoan on earth. Nematodes in several families are able to kill insects, known to be entomoparasitic nematodes. Of them, Mermithidae, Steinernematidae, and Heterorhabditidae have been more studied and some are considered as biological control agents for FAW (Huot et al., 2019; Ruiz-Nájera et al., 2013; Tarla et al., 2015; Viteri et al., 2018).

In general, mermithids nematode parasites can infect various hosts, such as spiders, mosquitoes, grasshoppers, or cockroaches (Košulič and Mašová, 2019; Kobylinski et al., 2012; Tarla et al., 2015). In addition, mermithids are also fatal to the insect host (Nikdel et al., 2011). Mermithid parasites have a great degree of species-specificity, so they are more promising to control target pests (Sáringer-Kenyeres et al., 2017). For example, in China the mermithid *Ovomermis sinensis* is the key mortality factors for *Mythimna separata* (Walker) (Sharma et al., 2002).

Recently, we found FAW naturally parasitized by a mermithid nematode in a field located in Hainan Province, China. This is the first report of *O. sinensis* (Nematoda: Mermithidae) parasitizing FAW *S. frugiperda* (Lepidoptera: Noctuidae) in the world. Our discovery can provide urgent and useful information on policy making for the control of FAW in China and Asia.

## Materials and methods

### Field survey

From May 8 to July 24, 2019, the collection of FAW was carried out in the corn fields of Qiongzhong County, Ledong County, Yazhou District, Danzhou City, and Qionghai City, Hainan Province. Large numbers of larvae were collected by a chessboard sampling method in maize fields in the above areas. The instar and number of FAW larvae obtained were recorded and all insects were brought back to the laboratory for feeding and observation. Digital images were obtained using Olympus BX63. For the molecular description, the nematodes were removed from the parasitized FAW.

### DNA extraction, PCR amplification, sequencing, and alignment

Genomic DNA was extracted from the nematodes using a Universal Genomic DNA Kit (CWBIO, China) according to the manufacturer’s protocol. Amplifications of 18 S and partial 28 S ribosomal DNA (D3 region) were performed according to Kobylinski et al. (2012) and Wang et al. (2007). As for 18 S, the following primers were used: 18S-F: 5’-CAAGGACGAAAGTTAGAGGTTC-3’ (forward) and 18S-R: 5’-GGAAACCTTGTTACGACTTTTA-3’ (reverse) (Kobylinski et al., 2012). A pair of primers of 28 S, 28S-5F: 5’-ACCCGTCTTGAAACACGGA-3’ (forward) and 28S-9R: 5’-TCGGAAGGAACCAGCTACTA-3’ (reverse) adopted from Wang et al. (2007) were used in D3 region study. Each polymerase chain reaction (PCR) was made in a total volume of 25 μl containing 12.5 μl × Es Taq MasterMix (CWBIO, China) 0.5 μl 10 μM of each primer, 2 μl template DNA and 9.5 μl ddH_2_O. PCR products were electrophoresed in 1.5% agarose gel, purified and sequenced in both directions with ABI 3730 (Suzhou Genewiz Biotechnology Co., Ltd., Tianjin, China). Sequences were aligned using Clustalw with the default settings in MEGA X software package (Kumar et al., 2018). Sequences were visually proofread, edited, and assembled into contigs in Bioedit v7.1.7 (Hall, 2012). The resulting sequences were submitted to GenBank. Sequences of mermithid from Genbank were searched and involved into the phylogenetic analysis. Pairwise distances and neighbor-joining (NJ) (Saitou and Nei, 1987) phylogenetic analysis were done using MEGA X software package (Kumar et al., 2018) under a Kimura 2-parameter (Kimura, 1980) model. Bootstrap analysis was computed with 500 replicates.

## Results

The prevalence of parasitic nematodes in parts of Hainan province is shown in Table 1. According to the survey data, the parasitic rate of the nematodes in FAW is about 2%. During the cultivation process, FAW was found and the survey distribution of nematode infection rate is shown in Table 1.

**Table 1.** Location, date, coordinates, and number of larvae, parasitic nematodes of fall armyworm (FAW) *Spodoptera frugiperda* and conditions of plants at three surveyed sites in Hainan province, China.

Date	Location	Coordinates	Total FAW (No.)	Parasitized FAW (No.)	Cornfield size (m^2^)	Plant height (cm)	Plant status	Planting gap (cm)	Spray insecticide situation
May 08, 2019	1 Wuna road, Qiongzhong Li and Miao autonomous county, Hainan Province, China	N 19°2´20˝ E 109°50´40˝	43	1	600	35	The late seedling	50	No spray insecticide
June 13, 2019	Qionghai X356, Hainan Province, China	N 19°15´51˝ E 110°28´49˝	52	1	2300	120	Jointing and booting stage	40	No spray insecticide
June 11, 2019	Foluo town, Ledong Li autonomous county, Hainan Province, China	N 18°61´97˝ E 108°73´38˝	105	2	990	150	Mature stage	45	No spray insecticide

### Diagnostic characters

**Figure 1:**
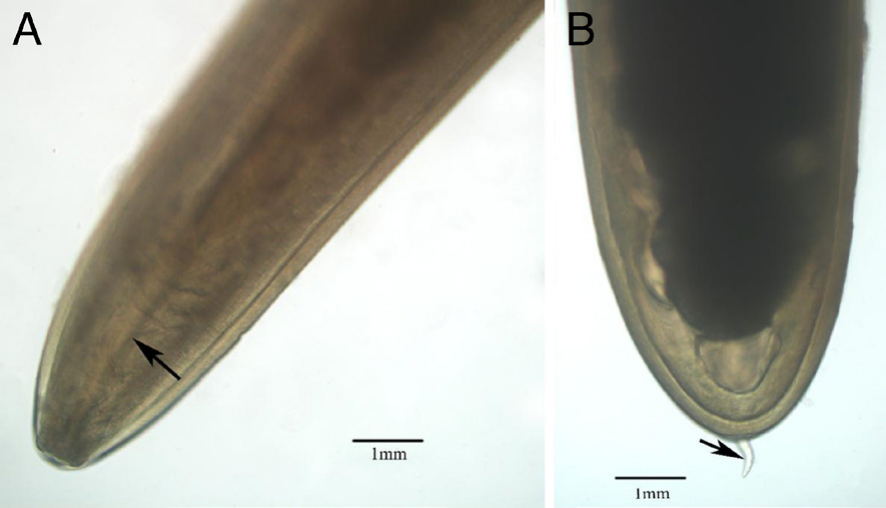
Microscopic photo of *Ovomermis sinensis*. A: Stylet on the anterior portion of post-parasitic juvenile (arrow); B: Tail appendage on post-parasitic juvenile (arrow).

**Figure 2:**
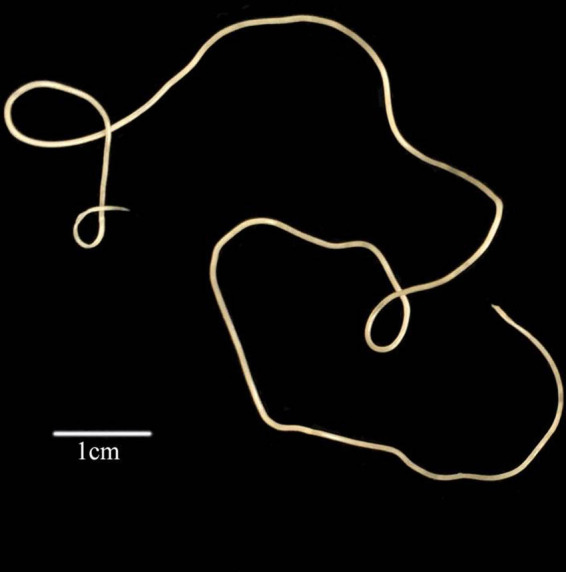
Post-parasitic juvenile *Ovomermis sinensis* nematode (scale bar: 1 cm).

**Figure 3:**
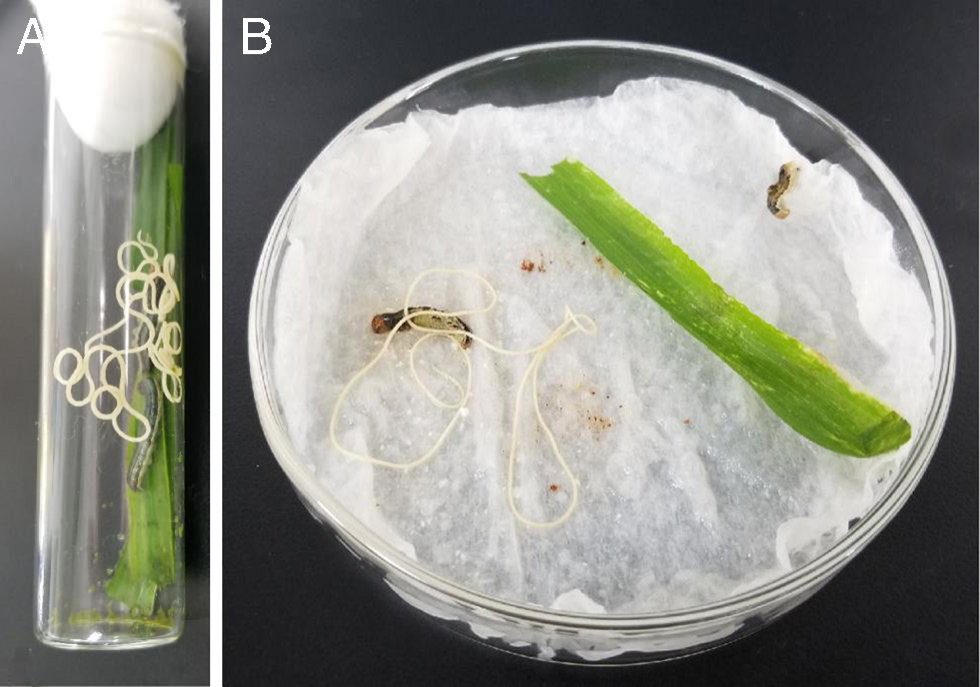
Post-parasitic juvenile *Ovomermis sinensis* nematode emerging from *Spodoptera frugiperda*.

According to morphological characteristics, these nematodes obtained from Hainan province belong to the family Mermithidae. This nematode has a stylet on the anterior portion of post-parasitic juvenile (Fig. 1A) and post-parasitic tail appendage (0.62 mm) on posterior end of post-parasitic juvenile (Fig. 1B). The mermithids are white and 228.5 mm in length (Fig. 2). *S. frugiperda* parasitized by the nematodes is shown in Figure 3.

### Molecular analyses

**Figure 4:**
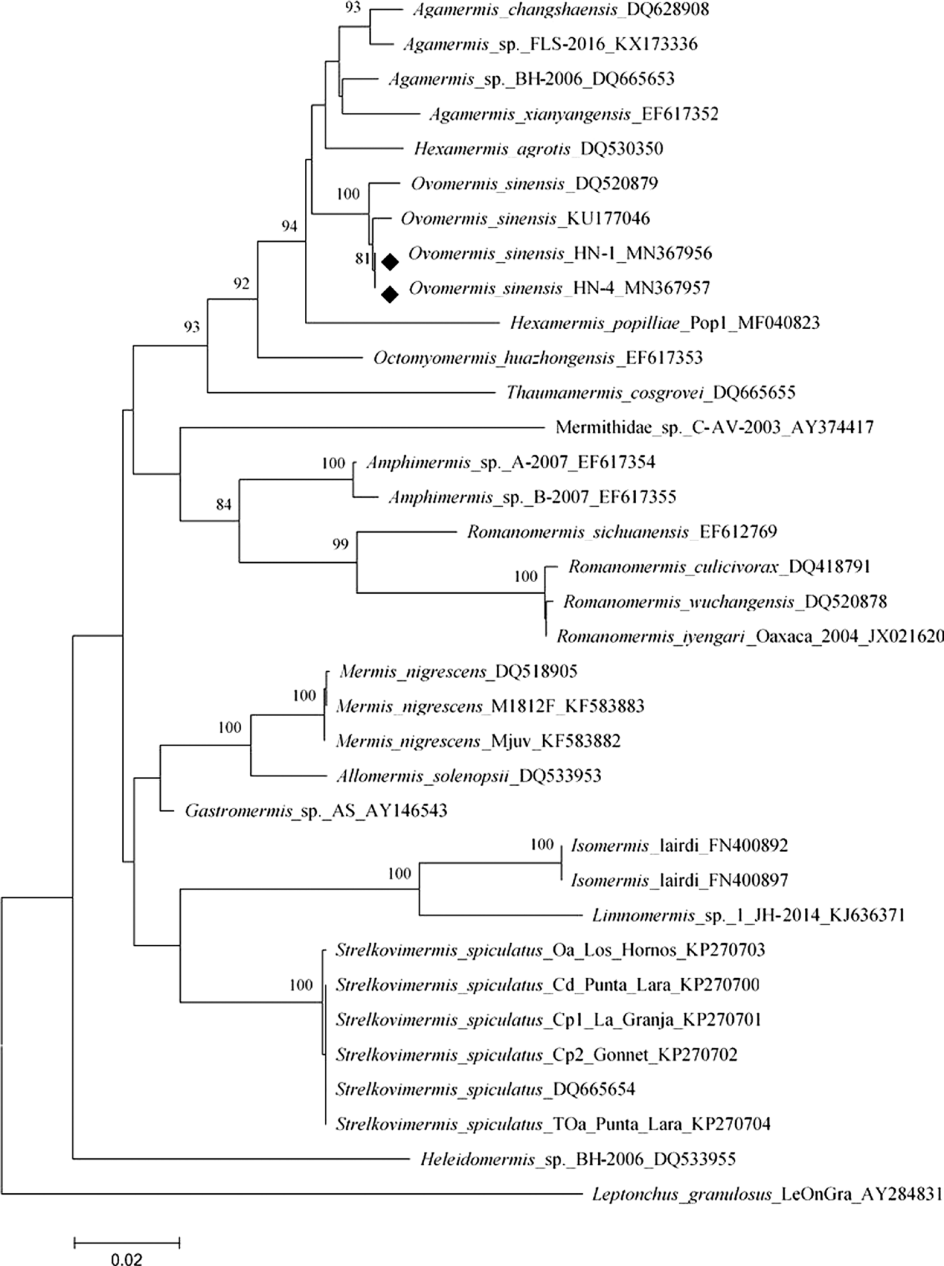
Neighbor-joining tree of the Mermithidae family. The tree was based on 18 S rDNA data and Kimura 2-parameter model; numbers on branches represent bootstrap support (>70%) based on 500 replicates; scale represents K2P genetic distance.

**Figure 5:**
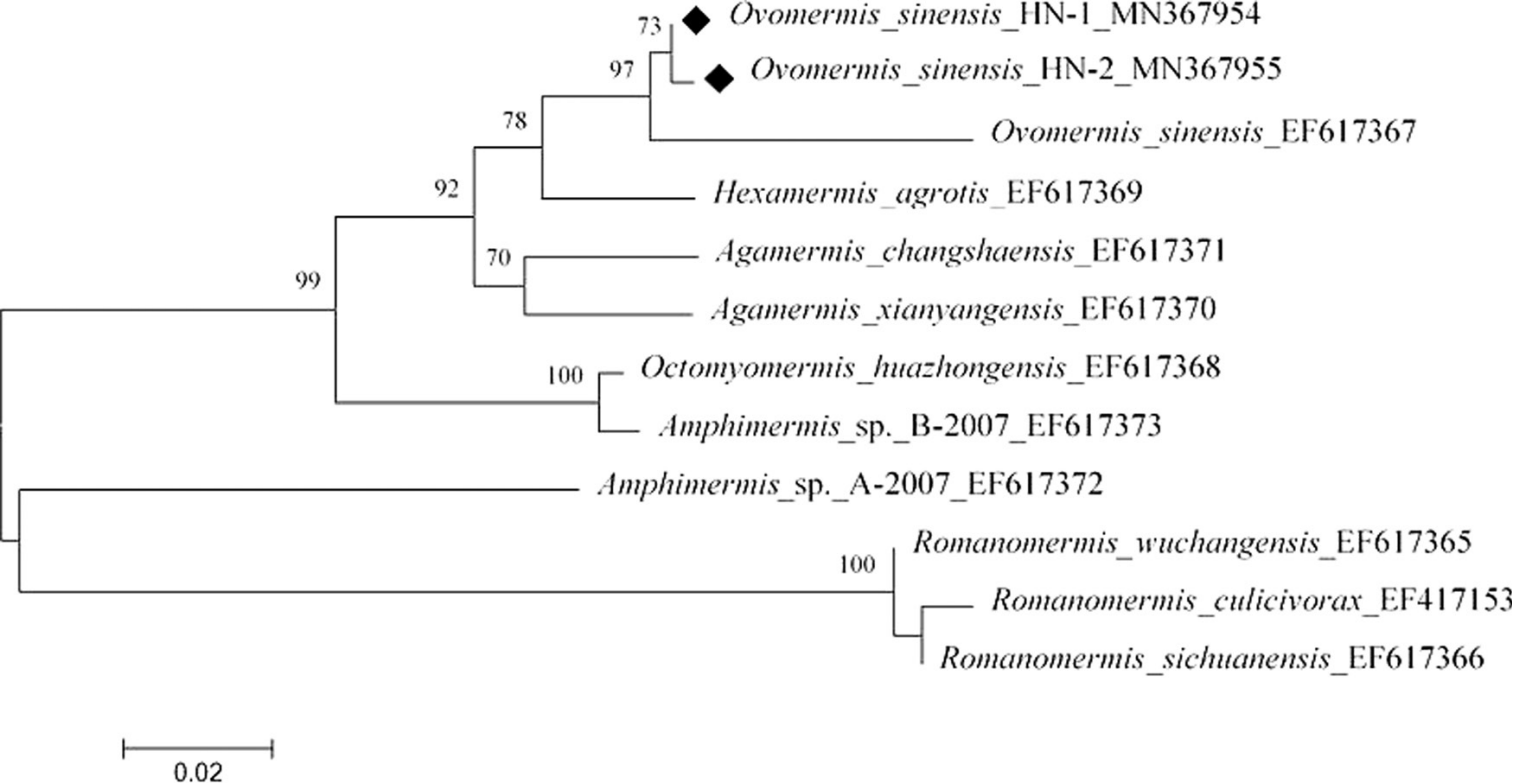
Neighbor-joining tree of the Mermithidae family. The tree was based on 28 S rDNA data and Kimura 2-parameter model; numbers on branches represent bootstrap support (>70%) based on 500 replicates; scale represents K2P genetic distance.

In the molecular analyses, the two individuals analyzed showed no polymorphism in the 18 S rDNA gene fragment detected (accession number MN367956 and MN367957). The D3 fragment of 28 S rDNA gene was uploaded with accession numbers MN367954 and MN367955. Based on the NJ trees of 18 S and D3 region in 28 S (Figs. 4 and 5), respectively, we confirmed that the nematodes isolated from *S. frugiperda* in Hainan province are *O. sinensis* and form an OTU with very small genetic distance.

**Figure S1:**
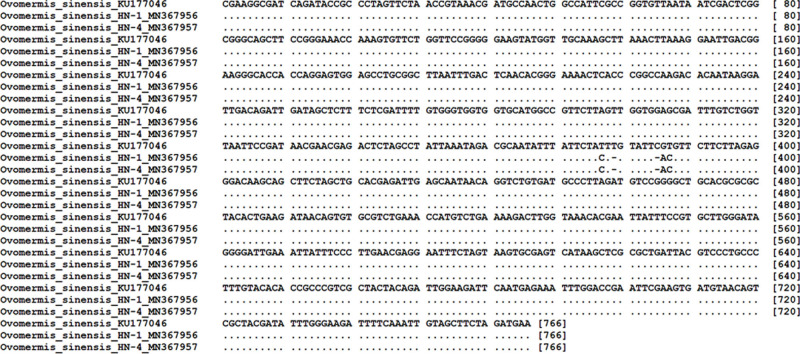
Alignment of 18 S sequences for comparative purposes of *Ovomermis sinensis* from Hainan province showed nucleotide identical to NCBI. Dashes are inferred insertion-deletion and dots indicate identity with the first sequence.

The 18 S rDNA sequences of HN-1 and HN-2 nematode specimens from Hainan Province were analyzed by Blast, and the similarity with *O. sinensis* (KU177046) was 99.35%. There are five base differences between them and the difference sites as shown in Figure S1. The corresponding genetic distance between the two tested samples (HN-1 and HN-2) and the known species *O. sinensis* from GenBank was 0.0040 and 0.0080 based on 18 S rDNA gene, 0.0836 and 0.0825 based on D3 region in 28 S rDNA gene, respectively. This genetic distance is significantly smaller than that between any two species, which is enough to suggest that the nematodes found in Hainan province belong to the species *O. sinensis*.

## Discussion

To our knowledge, this is the first report that *S. frugiperda* was parasitized by *O. sinensis* in a natural context, an indigenous mermithid nematode in China. In a survey conducted in Mexico, mermithid nematode species from another genus, *Hexamermis* sp., was found to be parasitic on FAW, causing a mortality rate of 8.42% (Ruiz-Nájera et al., 2013). The present study confirms new associations of mermithid nematodes *O. sinensis* as natural enemies of *S. frugiperda*.

*O. sinensis* had been efficiently used to control pest insects from family Noctuidae, i.e. *Spodoptera litura* and *Helicoverpa armigera* (Li et al., 2003) as well as *M. separata* (Walker) (Chen et al., 1991; Zhang et al., 1992). *S. frugiperda,* also from Noctuidae, was found naturally parasitized by *O. sinensis* in the preset study. All these findings confirmed the great potential of *O. sinensis* for control against the invasive pest insect *S. frugiperda* and other pest insects from Noctuidae. An intriguing finding in this study is that we can exploit the great potential to use *O. sinensis* to control FAW. Information on the occurrence and rates of parasitism of indigenous natural enemies is of paramount importance in designing a biological control program for *S. frugiperda*, either through conservation of native natural enemies or the introduction of new species for augmentative release. Major field releases of mermithids have been conducted for *Anopheles* population control (Kobylinski et al., 2012). Therefore, further study is needed to understand the biological and ecological relationship between *O. sinensis* nematodes and their hosts, and to explore the approach to artificially rear this nematode for potential field application in future.

## References

[ref001] Baudron F., Zaman-Allah M. A., Chaipa I., Chari N. and Chinwada P. 2019 Understanding the factors influencing fall armyworm (*Spodoptera frugiperda* J. E. Smith) damage in African smallholder maize fields and quantifying its impact on yield. A case study in Eastern Zimbabwe. Crop Protection 120:141–150.

[ref002] Chen G., Jian H., Ren H. F., Luo L. W., Zhou S. W., Zhang Z. L., Lei S. F. and Chen B. Q. 1991 Study on the natural control effect of *Ovomermis sinensis* on *Mythimna separata* . (in Chinese), Bulletin of Biological Control 7:145–150.

[ref003] Day R., Abrahams P., Bateman M., Beale T., Clottey V., Cock M., Colmenarez Y., Corniani N., Early R., Godwin J., Gomez J., Moreno P. G., Murphy S. T., Oppong-Mensah B., Phiri N., Pratt C., Silvestri C. and Witt A. 2017 Fall armyworm: impacts and implications for Africa. Outlooks on Pest Management 28:196–201.

[ref004] del Valle Loto F., Carrizo A. E., Romero C. M., Baigorí M. D. and Pera L. M. 2019 *Spodoptera frugiperda* (Lepidoptera: Noctuidae) strains from northern Argentina: esterases, profiles, and susceptibility to *Bacillus thuringiensis* (Bacillales: Bacillaceae). Florida Entomologist 102:347–352.

[ref005] Hall T. 2012 BioEdit version 7.1.7. available at: http://www.mbio.ncsu.edu/bioedit/bioedit.html (accessed November 25, 2012).

[ref006] Harrison R. D., Thierfelder C., Baudron F., Chinwada P., Midega C., Schaffner U. and van den Berg J. 2019 Agro-ecological options for fall armyworm (*Spodoptera frugiperda* J. E. Smith) management: providing low-cost, smallholder friendly solutions to an invasive pest. Journal of Environmental Management 243:318–330. 31102899 10.1016/j.jenvman.2019.05.011

[ref007] Huot L., George S., Girard P., Severae D., Negre N. and Duvic B. 2019 *Spodoptera frugiperda* transcriptional response to infestation by *Steinernema carpocapsae* . Scientific Reports 9:12789. 10.1038/s41598-019-49410-8 PMC6733877 31501491

[ref008] Kimura M. 1980 A simple method for estimating evolutionary rate of base substitutions through comparative studies of nucleotide sequences. Journal of Molecular Evolution 16:111–120. 7463489 10.1007/BF01731581

[ref009] Kobylinski K. C., Sylla M., Black W. and Foy B. D. 2012 Mermithid nematodes found in adult Anopheles from southeastern Senegal. Parasites & Vectors 5:131. 22741946 10.1186/1756-3305-5-131 PMC3439686

[ref010] Košulič O. and Mašová Š. 2019 First report on mermithid parasitism (Enoplea: Mermithidae) in a Southeast Asian spider (Araneae: Araneidae). Helminthologia 56:157–167. 31662687 10.2478/helm-2019-0012 PMC6799559

[ref011] Kumar S., Stecher G., Li M., Knyaz C. and Tamura K. 2018 MEGA X: molecular evolutionary genetics analysis across computing platforms. Molecular Biology and Evolution 35:1547–1549. 29722887 10.1093/molbev/msy096 PMC5967553

[ref012] Li C., Deng H., Wang G., Li T., Xu Y. and Gao Y. 2003 Appetite of *Spodoptera litura* larvae infected by *Ovomermis sinensis* . (in Chinese), Chinese Journal of Biological Control 19:205–205.

[ref014] Li X. J., Wu M. F., Ma J., Gao B. Y., Wu Q. L., Chen A. D., Liu J., Jiang Y. Y., Zhai B. P., Early R., Chapman J. W. and Hu G. 2019 Prediction of migratory routes of the invasive fall armyworm in eastern China using a trajectory analytical approach. Pest Management Science 76:454–463. 10.1002/ps.5530 31237729

[ref015] López M. A., Martínez-Castillo A. M., García-Gutiérrez C., Cortez-Mondaca E. and Escobedo-Bonilla C. M. 2018 Parasitoids and entomopathogens associated with fall armyworm, *Spodoptera frugiperda*, in Northern Sinaloa. Southwestern Entomologist 43:867–882.

[ref016] Nikdel M., Kaiser H. and Niknam G. 2011 First record of *Hexamermis cf. albicans* (Siebold, 1848) (Nematoda: Mermithidae) infecting Lepidopteran larvae from Iran. Nematologia Mediterranea 39:81–83.

[ref017] Ruiz-Nájera R. E., Ruiz-Estudillo R. A., Sánchez-Yáñez J. M., Molina-Ochoa J., Skoda S. R., Coutiño-Ruiz R., Pinto-Ruiz R., Guevara-Hernández F. and Foster J. E. 2013 Occurrence of entomopathogenic fungi and parasitic nematodes on *Spodoptera frugiperda* (Lepidoptera: Noctuidae) larvae collected in central Chiapas, México. Florida Entomologist 96:498–503.

[ref018] Saitou N. and Nei M. 1987 The neighbor-joining method: a new method for reconstructing phylogenetic trees. Molecular Biology and Evolution 4:406–425. 3447015 10.1093/oxfordjournals.molbev.a040454

[ref019] Sáringer-Kenyeres M., Kenyeres Z., Földvári G. and Majoros G. 2017 First record of mermithid larva (Nematoda: Mermithidae) in *Anopheles maculipennis* complex (Diptera: Culicidae) imago in Central-Europe. Biologia 72:1224–1227.

[ref020] Sharma H. C., Sullivan D. J. and Bhatnagar V. S. 2002 Population dynamics and natural mortality factors of the Oriental armyworm, *Mythimna separata* (Lepidoptera: Noctuidae), in South-Central India. Crop Protection 21:721–732.

[ref021] Shylesha A. N., Jalali S. K., Gupta A., Varshney R., Venkatesan T., Shetty P., Ojha R., Ganiger P. C., Navik O., Subaharan K., Bakthavatsalam N., Ballal C. R. and Raghavendra A. 2018 Studies on new invasive pest *Spodoptera frugiperda* (J. E. Smith) (Lepidoptera: Noctuidae) and its natural enemies. Journal of Biological Control 32:145–151.

[ref022] Souza M. L., Sanches M. M., de Souza D. A., Faria M., Espinel-Correal C., Sihler W. and Lopes R. B. 2019 Within-host interactions of *Metarhizium rileyi* strains and nucleopolyhedroviruses in *Spodoptera frugiperda* and *Anticarsia gemmatalis* (Lepidoptera: Noctuidae). Journal of Invertebrate Pathology 162:10–18. 30735762 10.1016/j.jip.2019.01.006

[ref023] Tarla G., Tarla S. and İslamoğlu M. 2015 First report of *Hexamermis* sp. (Nematoda: Mermithidae) parasitizing *Eurygaster maura* (Heteroptera: Scutelleridae) in an overwintering area. Florida Entomologist 98:974–978.

[ref024] Todd E. L. and Poole R. W. 1998 Keys and illustrations for the armyworm moths of the noctuid genus *Spodoptera* Guenée from the Western Hemisphere. Annals of the Entomological Society of America 73:722–738.

[ref025] Viteri D. M., Linares A. M. and Flores L. 2018 Use of the entomopathogenic nematode *Steinernema carpocapsae* in combination with low-toxicity insecticides to control fall armyworm (Lepidoptera: Noctuidae) larvae. Florida Entomologist 101:327–329.

[ref026] Wang J. Y., Xu F., Liu X. S. and Wang G. X. 2007 Molecular phylogeny of entomopathogenic nematodes (Mermithidae) inferred from DNA sequences of 18S rDNA, 28S rDNA and COI genes. Acta Zoologica Sinica 53:835–844.

[ref027] Yu S. J., Nguyen S. N. and Abo-Elghar G. E. 2003 Biochemical characteristics of insecticide resistance in the fall armyworm, *Spodoptera frugiperda* (J. E. Smith). Pesticide Biochemistry and Physiology 77:1–11.

[ref028] Zhang Z.-L., Lei S.-F., Wang H.-S., He J.-F. and Yang Y.-C. 1992 Release of the infective juveniles of *Ovomermis sinensis* (Nematoda: Mermithidae) to control armyworm, *Mythimna separata* (Lep.: Noctuidae) in wheat field in 1990. (in Chinese), Chinese Journal of Biological Control 8:148–150.

[ref029] Zhang L., Jin M. H., Zhang D. D., Jinag Y. Y., Liu J., Wu K. M. and Xiao Y. T. 2019 Molecular identification of invasive fall armyworm *Spodoptera frugiperda* in Yunnan Province. (in Chinese), Plant Protection 45:19–24.

[ref030] Zhao S., Sun X. X., Zhang H. W., Yang X. M. and Wu K. M. 2019 Laboratory test on the control efficacy of common chemical insecticides against *Spodoptera frugiperda* . (in Chinese), Plant Protection 45:10–14.

